# Supplementary material for: Limited protection against early-life lung murine cytomegalovirus infection results from deficiency of cytotoxic CD8 T cells
Source: PLoS Pathog. 2026 Apr 20;22(4):e1014150. doi: 10.1371/journal.ppat.1014150 (PMC13128127; doi:10.1371/journal.ppat.1014150)
Supplement: S1 Fig — Related to Fig 1. (A and B) (A) Representative flow cytometry plots and (B) pooled analysis of M25-specific CD4 T cells in the blood of infected and non-infected control mice. (C) Accumulation of NK cells, CD4 and CD8 T cells in the spleen (data as depicted in Fig 1F, left panel but with adjusted y-axis scale). Data display pooled results from 2 or more independent experiments (A and B, n = 2–14 per time point, C, n = 3–7 per time point). Lines in (B) indicate the median value. Numbers above each graph in (B) indicate the p value between adults and neonates of a 2-way ANOVA. (PDF) [file ppat.1014150.s002.pdf]

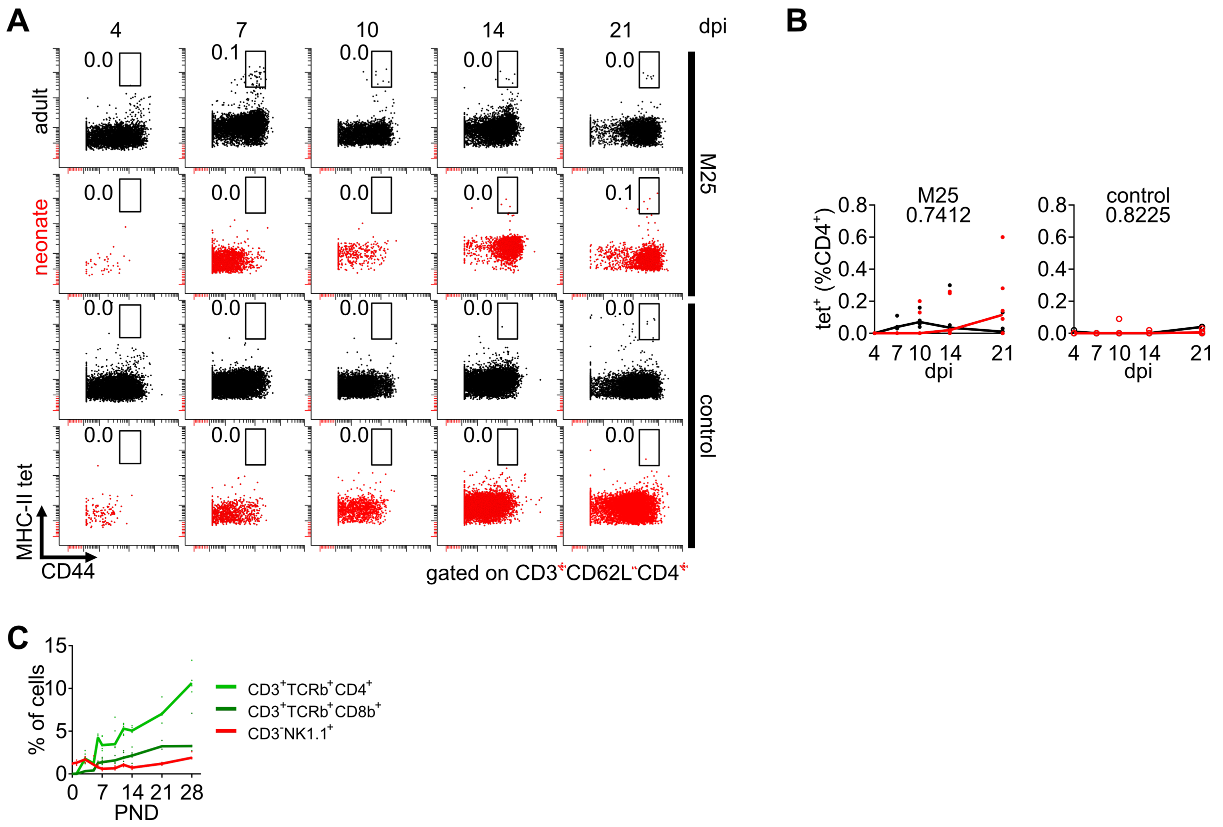

S1 Fig. Detection of MCMV-specific CD4 T cells and accumulation of T and NK cells in spleens of non-infected mice. Related to Fig 1.

(A and B) (A) Representative flow cytometry plots and (B) pooled analysis of M25-specific CD4 T cells in the blood of infected and non-infected control mice.

(C) Accumulation of NK cells, CD4 and CD8 T cells in the spleen (data as depicted in Fig 1F, left panel but with adjusted y-axis scale).

Data display pooled results from 2 or more independent experiments (A and B, n=2-14 per time point, C, n=3-7 per time point). Lines in (B) indicate the median value. Numbers above each graph in (B) indicate the p value between adults and neonates of a 2-way ANOVA.
