# Supplementary material for: Limited protection against early-life lung murine cytomegalovirus infection results from deficiency of cytotoxic CD8 T cells
Source: PLoS Pathog. 2026 Apr 20;22(4):e1014150. doi: 10.1371/journal.ppat.1014150 (PMC13128127; doi:10.1371/journal.ppat.1014150)
Supplement: S2 Fig — Related to Fig 2. (A) Quantitative analysis of CD44 expression on adoptively transferred lung eGFP+ T cells. Related to Fig 2D - 2G. (B) Quantitative analysis of lung immunohistology after adoptive transfer of adult polyclonal T cells. Related to Fig 2H. (C) Representative immunohistology of a neonatal lung NIF after MCMV-2DR infection and adoptive transfer of OT-I and OT-II cells. (D) Number of OT-I and OT-II cells in neonatal NIFs after MCMV-2DR or MCMV-4DR infection. (E) Correlation of number of OT-I cells per NIF with number of MCMV-4DR-infected cells per NIF in neonates. (E) NIF area and number of infected cells in NIFs of neonates infected with MCMV-4DR. Data display pooled results from 3 or more independent experiments (A, n = 5, B, n = 7–11 per time point, C-E, n = 3–4 per group). Numbers above each graph in (A), (C), and (E) indicate the p values of Mann-Whitney tests. (D) Nonparametric Spearman correlation r and two-tailed p value is provided. (PDF) [file ppat.1014150.s003.pdf]

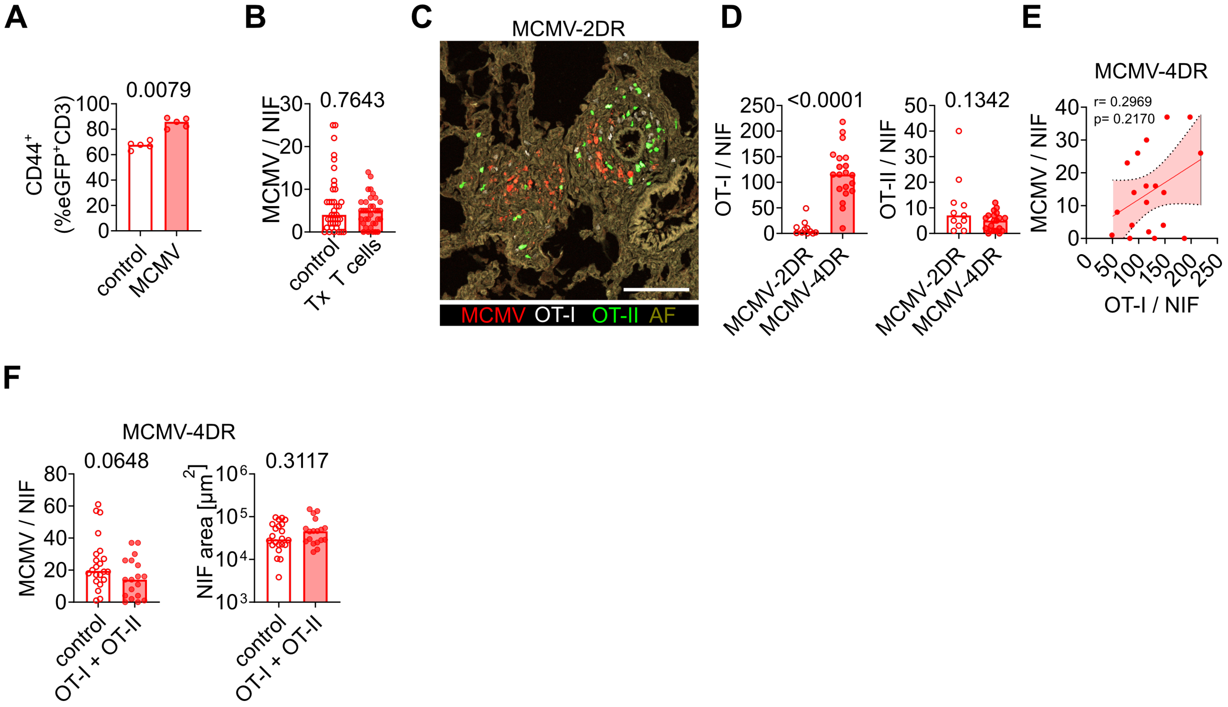


S2 Fig. Adoptive transfer of adult naïve T cells into neonates is not protective against MCMV. Related to Fig 2.

(A) Quantitative analysis of CD44 expression on adoptively transferred lung eGFP$^{+}$ T cells. Related to Fig 2D - 2G.

(B) Quantitative analysis of lung immunohistology after adoptive transfer of adult polyclonal T cells. Related to Fig 2H.

(C) Representative immunohistology of a neonatal lung NIF after MCMV-2DR infection and adoptive transfer of OT-I and OT-II cells.

(D) Number of OT-I and OT-II cells in neonatal NIFs after MCMV-2DR or MCMV-4DR infection.

(E) Correlation of number of OT-I cells per NIF with number of MCMV-4DR-infected cells per NIF in neonates.

(E) NIF area and number of infected cells in NIFs of neonates infected with MCMV-4DR.

Data display pooled results from 3 or more independent experiments (A, n=5, B, n=7-11 per time point, C-E, n=3-4 per group). Numbers above each graph in (A), (C), and (E) indicate the p values of Mann-Whitney tests. (D) Nonparametric Spearman correlation r and two-tailed p value is provided.
