# Supplementary material for: Limited protection against early-life lung murine cytomegalovirus infection results from deficiency of cytotoxic CD8 T cells
Source: PLoS Pathog. 2026 Apr 20;22(4):e1014150. doi: 10.1371/journal.ppat.1014150 (PMC13128127; doi:10.1371/journal.ppat.1014150)
Supplement: S3 Fig — Related to Fig 3. (A) Absolute numbers of analysed T cells in each group. (B) UMAP dimensionality reduction of CD4 T cells isolated from MCMV-infected animals. (C) Relative distribution of clonal sizes of all CD4 T cells. (D) Cytotoxicity module score of CD4 Th1 cells isolated from non-infected adults and neonates. (PDF) [file ppat.1014150.s004.pdf]

A

age	group	T cells	*Cd4+*	*Cd8a+*
adult	control	1978	609	852
adult	MCMV	1559	538	836
neonate	control	1266	463	529
neonate	MCMV	1220	404	688
	total	6023	2014	2905

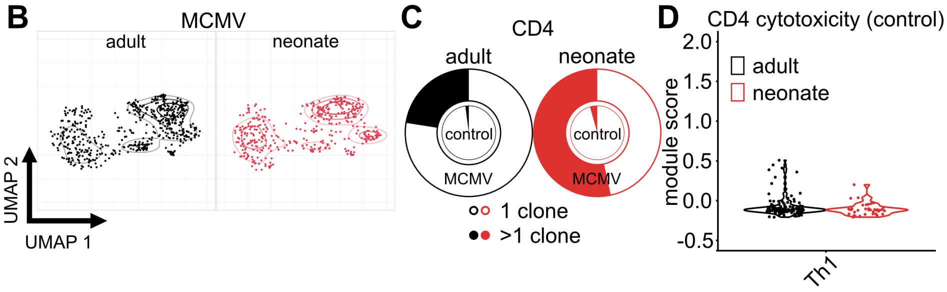

S3 Fig. Single-cell RNA sequencing of T cells primed in adult and neonatal mice. Related to Fig 3.

(A) Absolute numbers of analysed T cells in each group.

(B) UMAP dimensionality reduction of CD4 T cells isolated from MCMV-infected animals.

(C) Relative distribution of clonal sizes of all CD4 T cells.

(D) Cytotoxicity module score of CD4 Th1 cells isolated from non-infected adults and neonates.
