# Supplementary material for: Limited protection against early-life lung murine cytomegalovirus infection results from deficiency of cytotoxic CD8 T cells
Source: PLoS Pathog. 2026 Apr 20;22(4):e1014150. doi: 10.1371/journal.ppat.1014150 (PMC13128127; doi:10.1371/journal.ppat.1014150)
Supplement: S4 Fig — Related to Fig 4. (A) Analysis of CD44 protein expression by flow cytometry in adoptively transferred (eGFP+) or endogenous CD8+ T cells of neonates and adults, respectively. (B and C) (B) Representative flow cytometry plot and (C) pooled analysis of CXCR6 and CCL5 expression in adoptively transferred (eGFP+) or endogenous CD8+CD44+ T cells of neonates and adults, respectively. (D) Cytotoxicity module score of effector CD8 T cells isolated from non-infected adults and neonates. Data in A-C display pooled results from 2 independent experiments (n = 5–6). Numbers above each graph in (A) and (C) indicate the p values of 2-way ANOVAs. (PDF) [file ppat.1014150.s005.pdf]

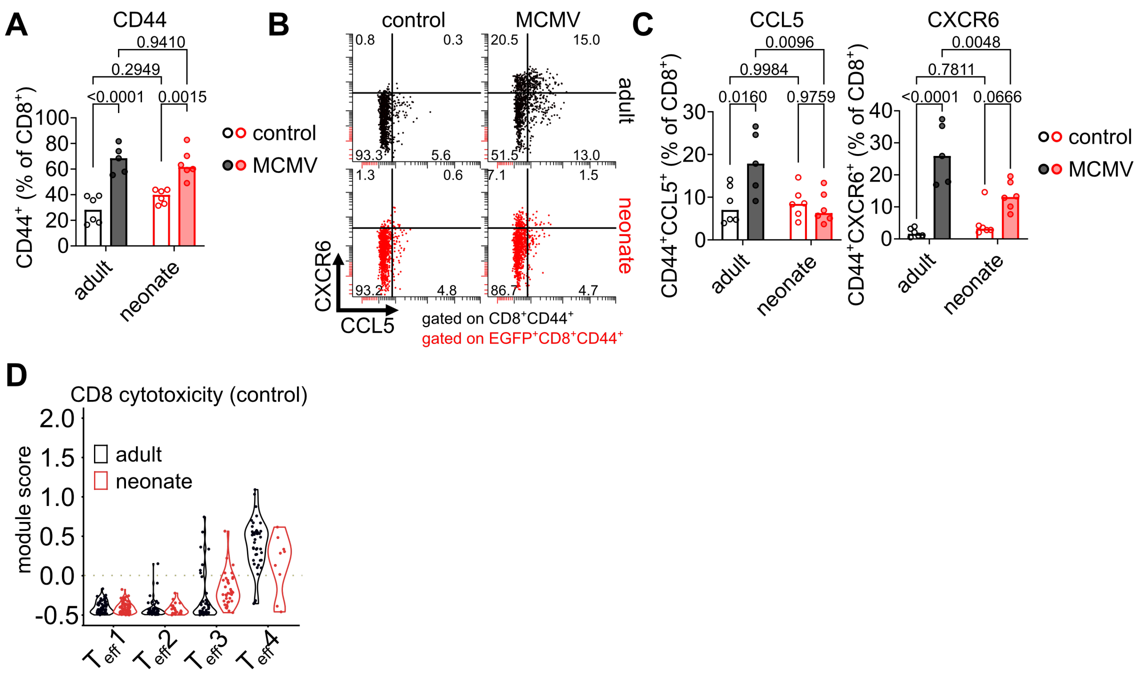

S4 Fig. Effector phenotype of CD8 T cells primed in adult and neonatal mice. Related to Fig 4.

(A) Analysis of CD44 protein expression by flow cytometry in adoptively transferred ($eGFP^+$) or endogenous $CD8^+$ T cells of neonates and adults, respectively.

(B and C) (B) Representative flow cytometry plot and (C) pooled analysis of CXCR6 and CCL5 expression in adoptively transferred ($eGFP^+$) or endogenous $CD8^+CD44^+$ T cells of neonates and adults, respectively.

(D) Cytotoxicity module score of effector CD8 T cells isolated from non-infected adults and neonates.

Data in A-C display pooled results from 2 independent experiments (n=5-6). Numbers above each graph in (A) and (C) indicate the p values of 2-way ANOVAs.
