# Supplementary material for: Limited protection against early-life lung murine cytomegalovirus infection results from deficiency of cytotoxic CD8 T cells
Source: PLoS Pathog. 2026 Apr 20;22(4):e1014150. doi: 10.1371/journal.ppat.1014150 (PMC13128127; doi:10.1371/journal.ppat.1014150)
Supplement: Attachment — Submitted filename: 260331_Point by point response.docx [file ppat.1014150.s009.docx]

**Additional Editor Comments:**

Dr. Stahl,
First of all let me begin by apologizing for the length of time it took to get reviews on your manuscript. Securing reviewers was a difficult process as invitees took days to inform us that they were too busy to provide a review and of the three reviewers we did secure, one failed to provide a review in a timely fashion in which their review was not received in over a month. I elected to drop them as a reviewer and make a decision with the two reviews we did receive. That being said, both reviewers were highly positive in terms of the quality and impact of your offering. Some concerns were raised (particularly with R1) which I believe can easily be addressed by your team. I anticipate the modified resubmission will be favorably recieved by the editorial staff.
sincerely, and again, I do apologize for the extended review time
Eain A. Murphy Ph.D.

We would like to thank the editor and reviewers for their detailed and insightful comments. We have carefully considered each recommendation, and the manuscript has greatly benefited from these suggestions. In the revised version, we have included modified text passages as well as additional experimental data to address the points raised. We are now confident that the manuscript, in its current form, meets the standards and expectations for publication in *PLOS Pathogens*.

**Reviewers' Comments:**
**Reviewer #1**

**Part I - Summary**

**Reviewer #1:** The authors provide convincing evidence for the delayed T cell response in MCMV-infected neonatal mice, and its implication in infection severity. The study uses various relevant and complementary approaches and techniques. Overall, the study is well done and convincing, and provides novel information that will be informative to the field. Some changes and corrections are however needed to improve the quality of the manuscript as currently presented.

**Part II – Major Issues**

**Reviewer #1:** 1) Fig1a (and following figures). The authors do not explain how the viral load was quantified. I assume by plaque assay here, and maybe via IVIS in Fig.2i. Please mention it in the figure legends and material and method.

We would like to thank the reviewer for pointing out the lack of methodological information. We used the recombinant MCMV-3DR, which encodes Gaussia luciferase, and tissue activity thereof allows for estimates of viral loads. We included this information and relevant references in the results and methods section.

It now reads:

Lines 81 - 85

**Delayed expansion of MCMV-specific CD8 T cells in early life**

To investigate the pathophysiology of the high early-life risk for CMV disease, we infected neonatal and adult mice by administering MCMV in the respiratory tract (13). We utilized an MCMV recombinant expressing mCherry and Gaussia luciferase that has previously been used in several in vivo studies to monitor and quantify MCMV infection (16-19).

Lines 466 - 471

**Determination of viral loads by MCMV-encoded luciferase activity**

Organs were homogenized with a TissueLyser II (Qiagen) machine in phosphate buffered saline, and MCMV-3DR-encoded Gaussia luciferase activity in organ supernatants after centrifugation was measured in duplicates after automatic addition of Coelenterazine (Synchem) with a Centro LB 960 XS3 microplate luminometer (Berthold). Luciferase values from non-infected animals were used as controls and to determine organ-specific limit of detection.

2) Fig1b-c (and S1a-b). n=3 mice per time point is low. An additional experiment with at least 3 more animals per time-point is needed to give more strength to the results.

We understand the concerns stated by the reviewer. While we carefully considered performing additional experimentation to increase the sample size already during the early phase of the project, we ultimately decided against it for several biological, ethical and logistical reasons.Expansion of MCMV-specific CD8 T cells has been explored elaborately by several groups in B6 mice [e.g. PMIDs: 26764033, 38619272, 31281313]. The present study builds up on a previous observation in which we already described a delayed accumulation of M45-specific CD8 T cells in neonates (PMID: 24348257). Using three MHC-I tetramers for detection of MCMV-specific CD8 T cells of different specificity, the data in the present manuscript now imply that accumulation of anti-MCMV specific CD8 T cells is generally delayed in neonates. We think that this is a result of the low T cell precursor frequency, which limits substantial CD8 T cells expansion in neonates. Moreover, the low numbers of alpha/beta T cells in the first days of life in mice impact the detection of antigen-specific cells simply due to the fact of low number of cells which can be analysed per animal. In fact, we analyzed one neonate for one MHC-I tetramer in independent experiments. While increasing the number of animals would further increase statistical power, the substantial effect size already achieved statistical significance. Considering the points mentioned above, we think performing additional experiments would not provide additional biological information. We were not authorized to perform such an extensive additional experimental series of 90 animals without submitting an amendment to the approved animal experimentation protocol. Moreover, these experiments need at least 8 weeks of time (2 weeks pre-mating stress reduction + 3 weeks pregnancy + 3 weeks post infection) to be performed. Together, additional experimentations would have substantially delayed the resubmission of the revised manuscript without adding substantial biological information. We therefore decided not to conduct additional animal experiments. In summary, we believe that the integration of our current data with previously published findings offers a clear and sufficient base for the delayed accumulation of MCMV-specific CD8⁺ T cells in early life.

The text now reads:

Lines 87 – 92

We detected CD8 T cells recognizing MCMV immunodominant epitopes M38, M45, and m139 (20) after seven days post-infection (dpi) in adults (Fig. 1B, C), consistent with previous reports (11, 21, 22). In neonates, MCMV-specific CD8 T cells could not be detected before 10 dpi, and the frequency of these cells was lower (Fig. 1B, C). This observation was in line with a previous study where we had observed a delayed expansion of M45 –specific CD8 T cells in neonatal mice (13).

3) “In contrast, there was a trend for more IL-10, IL-1β, and IFN-β (Fig. 5h-j) in neonates, and IFN-γ as well as IL-6 (Fig. 5k, l)…”. There is no statistical difference shown in Fig.5 between adult and neonates, not even a trend, regarding IL-10 and IL1b production during infection. Quite the opposite in fact, as IL10 and IL1b are present in non-infected adults, but not in neonates. Also, while the increase of IL-6 and IFNg seems to be true, can the authors explain/discuss why IL-6 and IFNg are decreased (even undetected) following infection in adult mice? Those values are somewhat unexpected. Is there not a group/condition inversion here in the way this result was presented? Finally, some numbers appear below the x-axis on Fig5.e, g, h, i, j, and k, and need to be removed.

We appreciate the reviewer’s comments and agree that the original manuscript text did not accurately reflect our observations. The text has been revised to clarify that, in early life, several cytokines became detectable only after MCMV infection, indicating a shift toward cytokines such as IL-10, IL-1β, IFN-β, IFN-γ, and IL-6. However, many of these measurements were close to or below the detection limit of the assay. Therefore, the immunoassay used may not have been sufficiently sensitive to fully resolve subtle differences in cytokine expression between the experimental groups. The numbers below the columns indicate the total number of mice analyzed, including samples in which cytokine levels were below the detection limit and therefore could not be graphically displayed.

We understand this reviewer’s question regarding a possible mix-up. After carefully re-examining our raw data and experimental documentation, we can confidently exclude a group swap. The multiplex nature of these experiments provides an internal control. While IFN-γ concentrations show the noted trend, the levels of the other cytokines measured simultaneously in these same samples were consistent with their assigned experimental groups. Although IFN-γ concentrations appeared lower in MCMV-infected adults compared to controls, this difference did not reach statistical significance. We therefore prefer not to speculate on trends that do not meet the predefined statistical threshold (p < 0.05).

We currently cannot conclusively explain why IL-6 concentrations were lower in MCMV-infected adults than in controls. As alveolar macrophages are a major source of IL-6 in the lung and known targets of MCMV infection, one possible explanation is that, at 5 dpi, cytopathic effects of the virus may impair their capacity to produce IL-6. In neonates, the early recruitment of myeloid cells into NIFs may compensate for the loss of alveolar macrophages more efficiently than adults. However, as we do not have experimental data to prove this hypothesis, we decided not to include speculative discussion of this point in the revised manuscript.

The text now reads:

Lines 209 - 224

**Ineffective early-life CD8 T cell immunity is associated with alterations in cytokine signaling conditions**

The effector phenotype of antiviral CD8 T cells is highly influenced by the inflammatory cytokine milieu within the infected tissue (28). Thus, we quantified cytokines known to be involved in CD8 T cell priming in lungs in steady state or in MCMV-mediated inflammatory conditions at 5 dpi. In general, more cytokines could be detected in non-infected adults than in neonates (Fig. 5A-L). MCMV infection induced a shift in the local adult cytokine environment to higher concentrations of Interleukin-2 (IL-2), IL-7, and IL-15, which was not detected in neonates (Fig. 5A-D). Moreover, there was an age-related difference for IL-27, IL-33, and Interferon-α (IFN-α), which were found in higher concentrations in adults (Fig. 5E-G). In neonates, IL-10, IL-1β, and IFN-β were detected in MCMV-infected but not control lungs, and there was no significant difference compared to cytokine concentrations in adults (Fig. 5H-J). IFN-γ as well as IL-6 were significantly higher in inflamed neonatal lungs than in controls (Fig. 5K, L). Adult T cells adoptively transferred into MCMV-infected neonates together with treatment of cytokine-antibody or -receptor complexes of IL-2 (29), IL-7, and IL-15 (30), but not cytokine complexes alone, reduced lung viral loads (Fig. 5M, N). Thus, there were remarkable age-related differences in signal 3 cytokines that likely impact T cell priming and effector differentiation.

Lines 838 - 846

**Fig. 5. Alterations in priming conditions in the early-life phase**

(A-L) Relative concentration of cytokines in steady state and MCMV-infected lungs at 5 dpi as indicated.

(M) Experimental setup for (N). MCMV-infected neonatal mice received 107 eGFP+ T cells and IL-2, IL-7, and IL-15 cytokine complexes or buffer control as indicated.

(N) Lung viral loads after adoptive T cell transfer and treatment with cytokines or buffer control.

Data acquired from two (A-L) or six (M-N) independent experiments. Values in A are shown on a normalized scale. Statistical differences were calculated with 2-way ANOVAs (B-L) or Mann-Whitney U test (N) and the p values are provided above the graphs. Numbers below columns of (C-K) indicated samples were the indicated cytokines was not detected (n.d.).

4) An important general comment is that by the time of infection, there are virtually no T cells in the neonates (Fig1e). One might argue that priming is not entirely the problem, and that the lack of T cells overall plays a major role. While the adoptive transfer experiments provide some clues to this question, T cells are transferred at the same time of the infection. At this time, the majority of these cells will not localized in secondary lymphoid organs, a likely site of their priming.
Did the authors ever infected pups at ~PND5-6, when endogenous T cells are present? Is there still a delay in tetramer+ T cells appearance? Would T cell have a decreased expression of CD44 at this time? Such a time-point is still very relevant to neonatal infection, as the 1st week of life in mice roughly match to the 3rd trimester of gestation in humans, especially with regards to immune system maturation.

The reviewer raises an important and highly relevant point, which we carefully considered during the early stages of the project design. In early life MCMV infection, there is T cell priming in secondary lymphoid organs and in NIFs. In a previous study, we found that adoptively transferred T cells migrated into NIFs with 24 hours (PMID: 24348257). In the present study, we found that adoptively transferred T cells substantially increase the absolute number of T cells in spleens 2 days after T cell transfer (see Figure 2 A-C). These data indicated that adoptively transferred T cells efficiently migrate into areas where antigen presenting cells are present. The expression of MCMV immediate early to late genes spans from approx. 4 to 24 hours. We estimate that by the time viral antigens are generated from active replication there should be sufficient numbers of T cells present at the affected anatomical regions. As both, the reference from a previous study as well as the additional information depicted in Figure 2 A-C of the present study are provided, we decided to not further discuss this point in the manuscript.

Unfortunately, we did not infect pups at ~PND5–6. While we agree that this time point is highly relevant to neonatal immunity there were both technical and biological limitations that prevented us from performing these experiments. Intratracheal MCMV infection of pups at PND5–6 is technically challenging, as animals at this stage are more developed, and performing a lung-targeted infection without anesthesia is likely not feasible. Currently, we do not have the authorization to perform such a procedure. We considered using an alternative MCMV application route (e.g. intraperitoneal) but this systematic application has a significant impact on the model system as viral antigens are distributed very fast into various organs. Most importantly, administration via this route differs fundamentally from intratracheal delivery. The respiratory tract acts as a highly effective barrier, permitting the use of substantially higher viral doses compared with the intraperitoneal route. The biological consequences of these differences for T cell priming are not well characterized and would require a series of carefully controlled experiments to dissect properly. We chose not to incorporate such extensive additional experimentation, as we believe it would go beyond the scope of the current manuscript and detract from its primary focus.

The text now reads:

Lines 111 - 115

In a previous study, we observed that adoptively transferred T cells migrated into neonatal lungs within 24 hours (13). Transferred cells homed to lymphoid organs and led to an approximately 60-fold increase of T cells in spleens within two days post intraperitoneal injection, reaching similar cell per body weight numbers as found in four-week-old mice (Fig. 2B, C).

**Part III – Minor Issues**

**Reviewer #1:** 1) Introduction: “…reasonable to assume that age-related differences in anti-CMV T cell immunity are responsible for the increased vulnerability in early life.". Involved, would likely be a more appropriate word. Most immune cells display drastic variation in neonates compared to adult mice, in number and/or phenotype.

Thank you. The text now reads:

Lines 53-57

CMV induces exceptionally strong T cell responses that are crucial for virus control and regularly lead to ~15 % of the circulating CD8 T cell pool being specific for CMV epitopes (8). Thus, it is reasonable to assume that age-related differences in anti-CMV T cell immunity are involved in the increased vulnerability in early life. Indeed, neonates exhibit an altered composition of peripheral blood T cell compartments and differential antigen-recognition patterns compared to adults (9).

2) Introduction: “The T cell effector phase itself was not disrupted indicating…”. Not clear what the authors refer to as effector phase. Priming is impaired, and with it, so are the effector functions of the T cells. This thought needs to be fleshed out.

Thank you for pointing this out. The text now reads:

Lines 71-79

Here, we delineate age-related differences in anti-MCMV T cell immunity. We demonstrate that the expansion of endogenous MCMV-specific T cells early in life is delayed due to an overall low frequency of peripheral T cells. Adoptive transfers of naïve adult T cells into neonates generated CD8 effector T cells that were low in cytotoxicity, and this correlated with their non-protective function in the young host. These non-cytotoxic CD8 T cells resulted from altered early-life T cell priming conditions. The impaired T cell response was not observed after adoptive transfer of effector T cells obtained from MCMV-infected adults, indicating that early life antiviral immunity was impaired at the level of T cell priming rather than the T cell effector phase. This study indicates that the early-life susceptibility to MCMV lung infection is linked to dampened cytotoxic T lymphocyte (CTL) induction.

3) FigS1a. m25-tetramer staining is not extremely convincing. This would benefit significantly from using double positive tetramer staining (e.g. Tet-APC+ Tet-PE+) to improve signal to noise ratio. Similar point for Fig2f (4 cells in the gate). This is likely not trivial in neonatal mice, but can the authors concatenate all of the samples from a group (on flowjo) to increase the number of cells shown in the dotplot/gate?

We thank the reviewer for the constructive feedback. A double-positive M25-tetramer staining would indeed likely increase the signal-to-noise ratio. We here provide a similar figure to Fig S1, but including the concatenated files for each group. As noted by the reviewer, there is a high background signal for the stainings with this tetramer, which led us to decide on setting the gate on the highly positive signals that should represent true M25-tetramer^+^ cells.

Flow cytometry plots of concatenated files from CD4+ T cells stained with M25 MHC-II tetramer.

We agree that neonatal lung analysis presents a significant technical challenge due to low absolute cell numbers. Regarding the suggestion to concatenate samples, while doing this in FlowJo would indeed increase the visual density of cells in the gate, we chose, throughout the manuscript, to present individual plots that represent the median value of the experimental group. We believe that this provides the most honest representation of the raw data. We also ensured that all gating is strictly validated against non-infected controls (or, when possible, FMOs) to confirm the accuracy of our populations despite the low event counts. We include here the dot plot of a sample with the most tetramer-positive cells to demonstrate the specificity of the tetramer staining after adoptive T cell transfer (see below).

Flow cytometry plots of tetramer stainings of the highest MHC-I tet^+^ sample from Fig 2G and a control sample from the same experiment.

4) Fig2d. “found the adult T cells to acquire CD44 membrane expression and proliferate”. While proliferation is undeniable, CD44 “acquisition” is (based on the provided plot). A large proportion of CD3+ cells are CD44+ (eF450+) in control animals. The authors need to either quantify and show total CD44+ cells (to prove their claim of increased expression of CD44) or rephrase to not be misleading.

We thank the reviewer for this suggestion. We have quantified the expression of CD44 on transferred eGFP+ adult T cells and included the data in Figure S2A.

The text now reads:

Lines 115-117

Next, we combined adoptive T cell transfers with MCMV infection and found the adult T cells to acquire CD44 membrane expression and proliferate in neonatal mice in response to virus challenge (Fig. 2D, e, and Fig S2A).

5) “… neonates can compensate for the deficiency of this cell population in the early-life period, lead to antigen-specific proliferation, gain of effector marker expression, homing into sites of MCMV infection, but has no influence on virus load in the lung.”. CD44 upregulation is not shown and likely weak. Without cytokines, cytotoxic molecules, or other membrane markers (41BB, OX40, CD25, CD69…), this is an overstatement.

We thank the reviewer for this comment. We added the increase of CD44 expression into Fig S2A and revised the text accordingly.

The text now reads:

Lines 138-141

The findings highlight that adoptive transfer of adult naïve T cells into neonates can compensate for the deficiency of this cell population in the early-life period, lead to antigen-specific proliferation, gain of CD44 expression, homing into sites of MCMV infection, but has no influence on virus load in the lung.

6) “However, there was a trend that adult T cells primed in MCMV infected neonates were more likely to differentiate into Th1 cells” Unclear why the authors make this claim, as the percentage of “Th1” is the same in both group during MCMV infection. In a more general way, it is unclear why the authors focus on CD4 here, as figure 1 and 2 clearly point toward CD8 T cells. Finally, these results suggest higher priming efficiency in neonates, directly contradicting their previous claim. Some discussion or clarification would be welcome here. The CD4 T cell transcriptomic analysis might be better suited after the CD8 transcriptomic analysis and kept in supplementary data.

We agree that the overall percentage of Th1 cells during MCMV infection appears comparable between the groups. Our intention was not to claim a statistically significant increase, but rather to note a trend suggesting a modest bias toward Th1 differentiation when adult naïve CD4 T cells are primed in the neonatal environment.

We agree that CD8 T cells are the principal effector population controlling MCMV infection. However, CD4 T cells are mechanistically highly relevant for several reasons. CD4 T cell help is essential for optimal CD8 T cell effector function and both cooperate to control MCMV in the lung (PMID: 30153311). Alterations in CD4 responses can directly influence CD8 T cell quality. In addition, CD4 regulatory T cells (Tregs) can suppress CD8 T cell immunity and may contribute to the generation of non-cytotoxic or functionally restrained CD8 T cells in early life. Furthermore, CD4 and CD8 T cell priming depend on partially distinct cytokine requirements and signaling pathways; therefore, findings in the CD4 compartment do not necessarily contradict our conclusions regarding CD8 T cell dysfunction. Our inclusion of CD4 analyses was thus hypothesis-driven, aiming to determine whether qualitative or regulatory differences in the CD4 compartment could help explain the altered CD8 phenotype observed in neonates.

The text now reads:

Lines 168-170

However, there was a trend suggesting that adult T cells primed in MCMV-infected neonates were likely to differentiate into Th1 cells (Fig. 3H), and these cells showed a distinct gene expression pattern when compared to Th1 cells found in adults (Fig. 3I, Table S1).

7) Fig4d. “CD62L-encoding gene Sell and differentiation-repressing genes Tcf7, Lef1, and Zeb1 were more abundant in neonates (Fig. 4d, Table S2).”. Please add gene names on the plot. Only Sell is shown.

We thank the reviewer for this suggestion and now have included these genes in the violin plot. Furthermore, to be consistent with the text we removed *Ctsd*, *Lgals3*, and *Ifitm*.

8) Fig4h. Teff1, which has the lower cytotoxic score, is the major effector cluster in neonates (before and after infection). Teff2, which proportion is similar in adult and neonates, show a decreased cytotoxic score in neonates. Teff3 and 4 (High cytotoxic score) are decreased in neonates. Data are convincing. The results description would benefit by rephrasing. Also, “…indicating that true CTLs are found within clusters”, this is only a transcriptomic data, not functional, please rephrase “true CTLs”.

The text now reads:

Lines 196-198

Next, module score analysis revealed that in adults, Teff2-4 but not Teff1 exhibited high cytotoxicity function in response to MCMV infection, indicating that antiviral CTLs are found within clusters Teff2-4 (Fig. 4I + Fig. S4D).

9) Fig6b ”At 8 dpi we found 1.9-fold more T cells in lungs”. Figure shows %, not absolute number. Need to rephrase, and/or to show the absolute number of T cells. “CD44 expression was higher on T cells in mice that received the adoptive transfers indicating that these cells had maintained their effector phenotype (Fig. 6d, e).” This is not actually shown. This claim implies increased MFI, which is not shown (and seems unchanged based on the plot shown). There is however an increase in the percentage of T cell expressing CD44.
The effect of the T cell transfer on the NIFs and viral load is very convincing.

The text now reads:

Lines 227-232

To confirm that T cells primed in adults are more protective against MCMV we isolated CD44^+^ T cells from infected adult lungs and adoptively transferred these into neonatal mice (Fig. 6A). At 8 dpi we found a 1.9-fold higher frequency of T cells in lungs of mice that received adult effector T cells as compared to mice without cell transfer (Fig. 6B, C). The frequency of CD44^+^ cells was higher on T cells in mice that received the adoptive transfers, indicating that these cells had maintained their effector phenotype (Fig. 6D, E).

An additional informative control here would be to inject effector T cell from non-MCMV infected mice. In other words, is the effector T cell transfer efficient at reducing viral load due to bystander action, or is it by MCMV-specific T cells? Did the authors stain transferred CD44+ T cell with tetramers before transfer and at endpoint? The authors could also get at this issue by transfering pre-activated OT-I and OT-II, and infect with a WT virus (not expressing OVAp)

We thank the reviewer for this suggestion. We decided to include an additional control by adoptively transferring purified CD44+ T cells obtained from non-infected adult mice into neonates. This treatment had no impact on viral loads in neonatal lungs. The data is now included in the Fig 2J.

The text now reads:

Lines 123 – 124

Transfer of purified CD44+ T cells from adult non-infected mice also did not protect against MCMV (Fig. 2J).

10) Discussion.
“Murine respiratory tract Cd44-, Gzmb-, and Ccl5-expressing CD8 effector T cells encompassed four subpopulations that differed in their expression of effector molecules.” Needs rephrasing here. Those markers are not the one allowing sub-selection of total CD8 effector T cells. Indeed as per Fig4b and g, only a fraction of effector CD8 expresses GzmB and CD44. “…and those cells that clustered as Teff2 exhibited..” please rephrase.

We thank the reviewer for the suggestion. We have classified the effector CD8 T cells according to previously published data as mentioned in the manuscript (PMID: 32846135 and 32313720). Though these were detected as marker genes in these clusters, it is true that not all cells in the effector cluster showed a high combined expression of Cd44, Gzmb, and Ccl5, particularly in neonatal T_eff_2. Accordingly, we modified the text. The text now reads:

Lines 245-246

Murine respiratory tract CD8 effector T cells encompassed four subpopulations that differed in their expression of additional effector molecules.

“Accordingly, effector CD8 T cells isolated from adults efficiently reduced MCMV loads in neonates arguing that the CD8 T cell effector function is not disrupted in the young host.” This is not clear. It seems the authors want to say that there are no inhibitory mechanisms or pathway in neonates that would inhibit pre-existing effector function from the adult CD8 T cells. “Instead, CD8 T cell priming in early life impairs their differentiation program into cytotoxic CTLs”. It is likely that the priming itself is impaired, leading to defective differentiation

We thank the reviewer for indicating that precise wording is needed to deliver our thought to the reader as intended.

The text now reads:

Lines 256-261

Accordingly, effector CD8 T cells isolated from adults efficiently reduced MCMV loads in neonates. These data indicate that potential inhibitory mechanisms in neonates that would interfere with the function of adoptively transferred antiviral effector CD8 T cells are not prominent during the T cell effector phase in the young host. Instead, CD8 T cell priming in early life impairs their differentiation program into cytotoxic CTLs and increases their vulnerability to infection.

**Reviewer #2**

**Part I – Summary**

**Reviewer #2:** In this study, Brito et al examine the mechanisms that make mice susceptible to lung disease caused by infection with the murine cytomegalovirus (MCMV) in the early stages of life. Through a series of well-executed experiments, including adoptive T-cell transfer, the researchers convincingly demonstrate that defective cytokine-mediated priming of MCMV-specific CD8+ T cells in neonates is a key factor for the increased risk of viral lung infection in early life.

The work is presented almost flawlessly, and all conclusions appear to be justified by the provided data.

**Part II – Major Issues**

**Reviewer #2:** My only concern about this study is that the authors could further clarify what significant advances they are presenting compared to the existing literature. While the results are convincing and well presented, the findings are unsurprising and offer limited novelty.

We thank Reviewer for this thoughtful comment. We respectfully submit that the principal advance of our study lies not in the description of a single unexpected mechanism, but in the systematic and comprehensive in vivo dissection of early-life antiviral T cell immunity in the context of MCMV infection.

In this project, we addressed several fundamental and interrelated questions:

- What are the kinetics of MCMV-specific T cell responses in early life?
- Can adult naïve T cells differentiate into functional antiviral effector T cells in a neonatal environment?
- How does early-life priming shape the effector phenotype of CD8 T cells?
- Which mechanisms contribute to the generation of non-cytotoxic CD8 T cells in early life?
- Is there a distinct (potentially regulatory) CD4 T cell response in early life that could influence CD8 T cell functionality?
- Is the defective antiviral response primarily determined at the level of T cell priming, or does impairment occur at the effector stage?

To our knowledge, these questions have not previously been addressed in such an integrated and comparative manner in vivo. Importantly, several aspects of the dataset were unexpected to us, particularly the dissociation between T cell expansion and acquisition of cytotoxic function, and the differential contribution of priming versus effector-stage regulation.

We agree with the reviewer that our findings may not reveal a single unifying mechanism. However, we believe that this is precisely an important conceptual advance: early-life antiviral T cell immunity is not explained by a simple intrinsic defect, but rather reflects a complex interplay of developmental, environmental, and regulatory factors. By systematically dissecting these components–and by transparently presenting both positive and negative findings–we provide a framework that helps refine prevailing assumptions in the field.

We therefore believe that the novelty of this work lies in its comprehensive in vivo analysis and in the clarification of where early-life T cell immunity is, and where it is not, fundamentally impaired. We hope this perspective better highlights the conceptual advances of the study. To clarify the advance of this study we added a paragraph summarizing the key findings.

The text now reads:

Lines 320 -327

In summary, we investigated the kinetics of MCMV-specific T cell responses in early life by delineating the expansion of endogenous CD8⁺ T cells and assessing the impact of adoptive transfer of naïve adult T cells into neonates. We characterized the mechanisms underlying the generation of non-cytotoxic CD8⁺ T cells, as well as whether impaired antiviral responses arise during priming or at the effector stage. Our data indicate that deficiencies in inflammatory cytokines during neonatal priming drive the emergence of non-cytotoxic CD8⁺ T cells, whereas adult-primed T cells retain full effector functionality in neonatal hosts, identifying the priming environment as the key determinant of the impaired response.

**Part III – Minor Issues**

**Reviewer #2:** The manuscript is well written, with only the occasional typo (e.g. Author Summary: 'effector T cells obtain from adult hosts'; Results: 'there were remarkable differences in signal 3 cytokines'). These errors should be corrected.

We thank the reviewer for this comment. We have proofread the manuscript for typos.

**References**

1.	Lueder, Y., et al., *Control of primary mouse cytomegalovirus infection in lung nodular inflammatory foci by cooperation of interferon-gamma expressing CD4 and CD8 T cells.* PLoS Pathog, 2018. **14**(8): p. e1007252.

2.	Marquardt, A., et al., *Single cell detection of latent cytomegalovirus reactivation in host tissue.* J Gen Virol, 2011. **92**(Pt 6): p. 1279-1291.

3.	Bosnjak, B., et al., *MCK2-mediated MCMV infection of macrophages and virus dissemination to the salivary gland depends on MHC class I molecules.* Cell Rep, 2023. **42**(6): p. 112597.

4.	Jordan, S., et al., *Virus progeny of murine cytomegalovirus bacterial artificial chromosome pSM3fr show reduced growth in salivary Glands due to a fixed mutation of MCK-2.* J Virol, 2011. **85**(19): p. 10346-53.

5.	Arutyunyan, A., et al., *Spatial multiomics map of trophoblast development in early pregnancy.* Nature, 2023. **616**(7955): p. 143-151.

6.	Qin, Y., et al., *Selective knockout of key CMV receptors in fetal cells blocks direct and endocytic pathways of entry in the guinea pig.* bioRxiv, 2025.

7.	Bowen, J.A. and J.S. Hunt, *Expression of cell adhesion molecules in murine placentas and a placental cell line.* Biol Reprod, 1999. **60**(2): p. 428-34.

8.	Lane, R.K., et al., *Necroptosis-based CRISPR knockout screen reveals Neuropilin-1 as a critical host factor for early stages of murine cytomegalovirus infection.* Proc Natl Acad Sci U S A, 2020. **117**(33): p. 20109-20116.

9.	Kawasaki, H., et al., *Cytomegalovirus initiates infection selectively from high-level beta1 integrin-expressing cells in the brain.* Am J Pathol, 2015. **185**(5): p. 1304-23.

10.	Sitnik, K.M., et al., *Fibroblasts are a site of murine cytomegalovirus lytic replication and Stat1-dependent latent persistence in vivo.* Nat Commun, 2023. **14**(1): p. 3087.

11.	Marsh, B. and R. Blelloch, *Single nuclei RNA-seq of mouse placental labyrinth development.* Elife, 2020. **9**.

12.	Wagner, M., et al., *Systematic excision of vector sequences from the BAC-cloned herpesvirus genome during virus reconstitution.* J Virol, 1999. **73**(8): p. 7056-60.

13.	Stahl, F.R., et al., *Mck2-dependent infection of alveolar macrophages promotes replication of MCMV in nodular inflammatory foci of the neonatal lung.* Mucosal Immunol, 2015. **8**(1): p. 57-67.
